# From mitochondria to heart: the role and challenges of mitochondrial antiviral signaling protein in cardiovascular disease

**DOI:** 10.3389/fcvm.2025.1572559

**Published:** 2025-08-06

**Authors:** Mengting Jiang, Runze Huang, Wei Li

**Affiliations:** ^1^ The key Laboratory of Myocardial Remodeling Research, The Affiliated Hospital of Guizhou Medical University, Guiyang, Guizhou, China; ^2^ Department of Cardiovascular Medicine, The Affiliated Hospital of Guizhou Medical University, Guiyang, Guizhou, China

**Keywords:** mitochondrial antiviral signaling protein (MAVS), inflammation, mitochondrial homeostasis, innate immunity, cardiovascular diseases

## Abstract

Mitochondrial Antiviral Signaling Protein (MAVS) is a pivotal adaptor protein in the innate immune response, mediating the activation of NF-κB and type I interferon signaling pathways during viral infections. As an integral component of the mitochondrial outer membrane, MAVS also plays critical roles in the regulation of apoptosis, cellular metabolism, and the activation of inflammasomes, including NLRP3 and caspase family members. Emerging evidence indicates that MAVS is not only essential in antiviral defense but also contributes significantly to the pathogenesis of various diseases, notably cardiovascular diseases. In this review, we provide a comprehensive overview of the molecular structure of MAVS and the regulatory mechanisms modulating its activity. We further highlight the involvement of MAVS in the development of cardiovascular diseases through its participation in innate immune signaling and mitochondrial dynamics. Particular attention is given to the regulation of MAVS by post-translational modifications—such as ubiquitination, methylation, and acetylation—as well as by microRNAs and other mitochondria-associated proteins. These insights aim to deepen the understanding of MAVS as a potential biomarker and therapeutic target, offering novel perspectives for the prevention, diagnosis, and immunotherapeutic intervention of cardiovascular diseases.

## 1 Introduction

Cardiovascular diseases are one of the leading causes of death and disease burden worldwide, with complex and diverse pathogenesis, including various pathological processes such as inflammatory response, lipid metabolism disorders, and apoptosis. Mitochondria are double-membrane organelles found in mammalian cells that can regulate and respond to different stress sources and metabolic demands, enabling them to effectively coordinate various cellular functions. In recent years, with the advancement of research, the critical role of mitochondria in the occurrence and development of cardiovascular diseases has gradually drawn attention (1).

MAVS is a key receptor of the innate immune system primarily located on the outer membrane of mitochondria. MAVS senses viral invasion and activates downstream signaling pathways, such as NF-κB and IRF3, thereby promoting the production of type I interferons (I-IFN) and pro-inflammatory cytokines, further participating in antiviral immune responses. Emerging evidence suggests that MAVS is also closely related to the pathogenesis of various cardiovascular diseases.

This review aims to summarize the mechanism of action of MAVS in cardiovascular diseases and its potential clinical application value. We will first review the basic functions of MAVS in innate immunity, then conduct an in-depth analysis of the specific roles of MAVS in different types of cardiovascular diseases, including viral myocarditis, heart failure, and myocardial infarction. By systematically summarizing the research progress of MAVS in the cardiovascular system, we aim to offer new insights into the prevention and treatment of cardiovascular diseases in the future.

## 2 Molecular structure and biological functions of MAVS

### 2.1 Genes and protein structure of MAVS

The MAVS gene, located on human chromosome 20p13, encodes a polypeptide chain composed of 540 amino acids. The MAVS protein is mainly divided into three domains, each playing a crucial role in the function of MAVS.

The N-terminal Caspase Activation and Recruitment Domain (CARD) containing cysteine aspartate protease: The main function of this domain is to interact with the CARD domains of Retinoic Acid-Inducible Gene I (RIG-I) and Melanoma Differentiation-Associated Gene 5 (MDA5), thereby initiating the antiviral signaling cascade. The interaction of the CARD domain is a key step in MAVS signal transduction, determining its responsiveness to viral infections. In addition, the CARD domain of MAVS participates in activating caspase proteins and the NLRP3 inflammasome, mediating its own cleavage to regulate immune homeostasis (2, 3).

The PRR Domain (Proline-Rich Region) in the middle: the PRR domain contains a TRAF interaction motif (TIM) and a proline-rich domain (PRD). TIM allows MAVS to interact with various tumor necrosis factor receptor-associated factors (TRAF) family proteins, promoting downstream signaling, while PRD acts as a scaffold for recruiting E3 ubiquitin ligase, playing a key role in the activation and regulation of immune responses mediated by MAVS.

**Figure 1:**
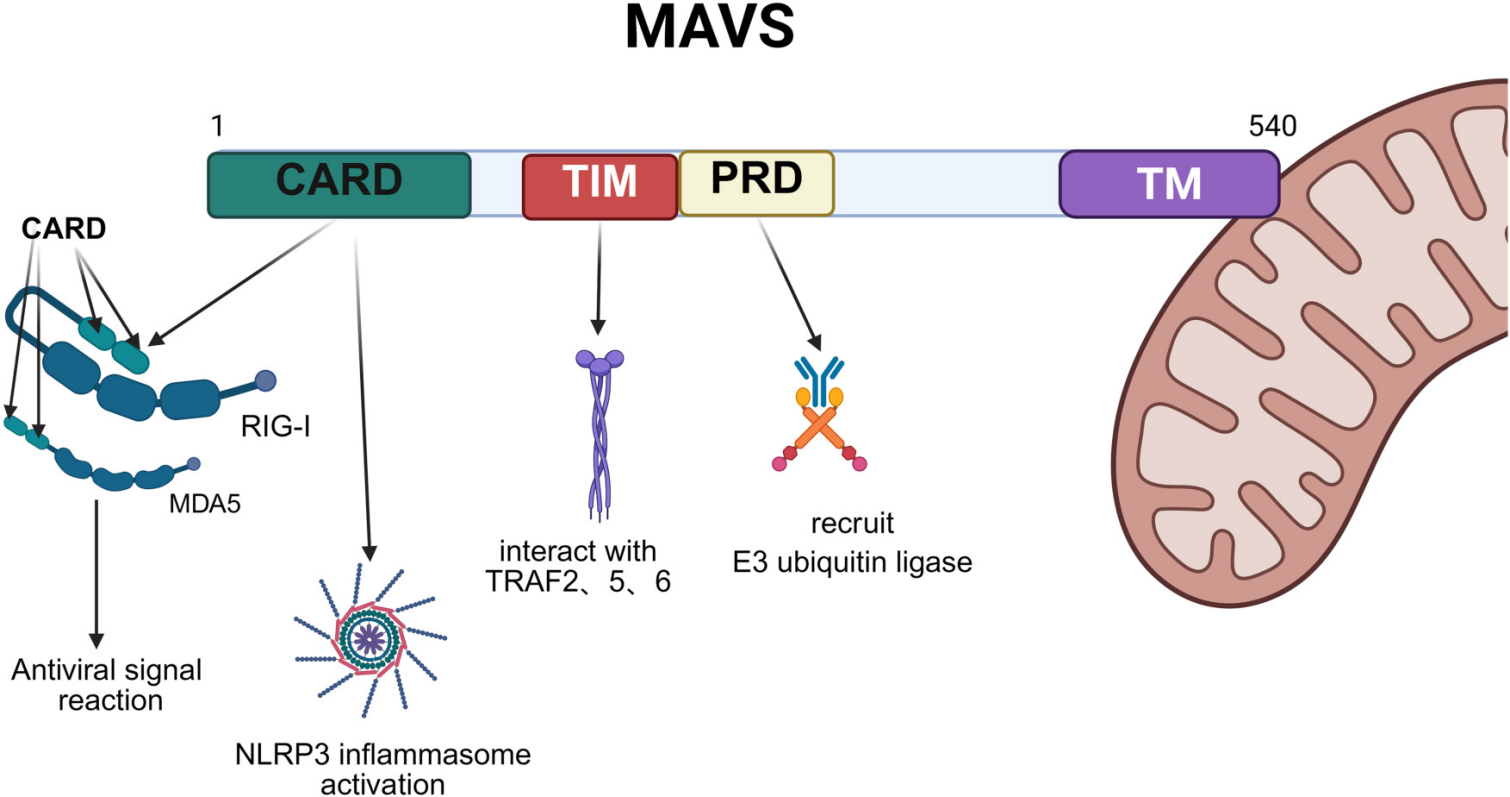
Represented illustration of the main domains of MAVS. CARD domain (green) of MAVS interacts with the CARD domains of RIG-I and MDA5, thereby initiating the antiviral signaling cascade; TIM (red) in the PRR domain interacts with TRAF family members, activating the IKK and TBK-1 complex. PRD (yellow) participates in recruiting E3 ubiquitin ligase; TM (purple) anchors MAVS to the outer membrane of mitochondria, ensuring its stability and effectiveness. Created with Biorender.com.

The C-terminal TM (Transmembrane) domain: This domain anchors MAVS to the outer membrane of mitochondria, ensuring its stability and effectiveness in antiviral signaling. The presence of this domain enables MAVS to transduce signals from the viral sensor RIG-I-like receptors (RLR) pathway to downstream effectors. In addition, the TM domain also facilitates the aggregation of MAVS on the membrane, forming inflammasomes that contain TRAF3, TRAF6, and other signaling molecules, further amplifying the antiviral signal (4) (Figure 1).

### 2.2 Mechanisms of innate immunity involving MAVS and its distribution

When facing bacterial or viral infection, MAVS initiates downstream signaling cascades by interacting with the CARD domains of RIG-I or MDA5 (5). The specific process involves RIG-1 and MDA5 undergoing conformational changes upon contact with viral DNA or RNA, exposing their N-terminal CARD domains to form tetramers. The E3 ubiquitin ligase modifies the CARD with polyubiquitination, thereby promoting the binding of RIG-1 and MDA5 to MAVS through the CARD. MAVS forms prion-like protease-resistant fibrils that convert other MAVS on the mitochondrial outer membrane into prion-like aggregates (6). Prion-like aggregates are the basis for antiviral immune defense and inflammasome activation signal transduction.

Subsequently, MAVS interacts with TRAF2, TRAF3, TRAF5, or TRAF6 to promote the activation of the TBK1 complex (TANK-binding kinase 1) (containing TBK1, i-κb kinase (IKK)i/ε and NEMO) in the presence of TRAF2/3/5/6, and to promote the activation of the IKK complex (containing IKKα/β and NEMO) in the presence of TRAF2/5/6. The TBK1 complex promotes the phosphorylation of IRF3 and/or IRF7, leading to nuclear translocation and binding to the IFN-stimulated response element, thereby inducing the transcription of target genes. Similarly, the TRAF2/5/6-activated IKK complex activates NF-κB, promoting the transcription of pro-inflammatory cytokines. Therefore, the two MAVS-mediated signaling pathways play different but crucial roles in antiviral innate immunity (4).

**Figure 2:**
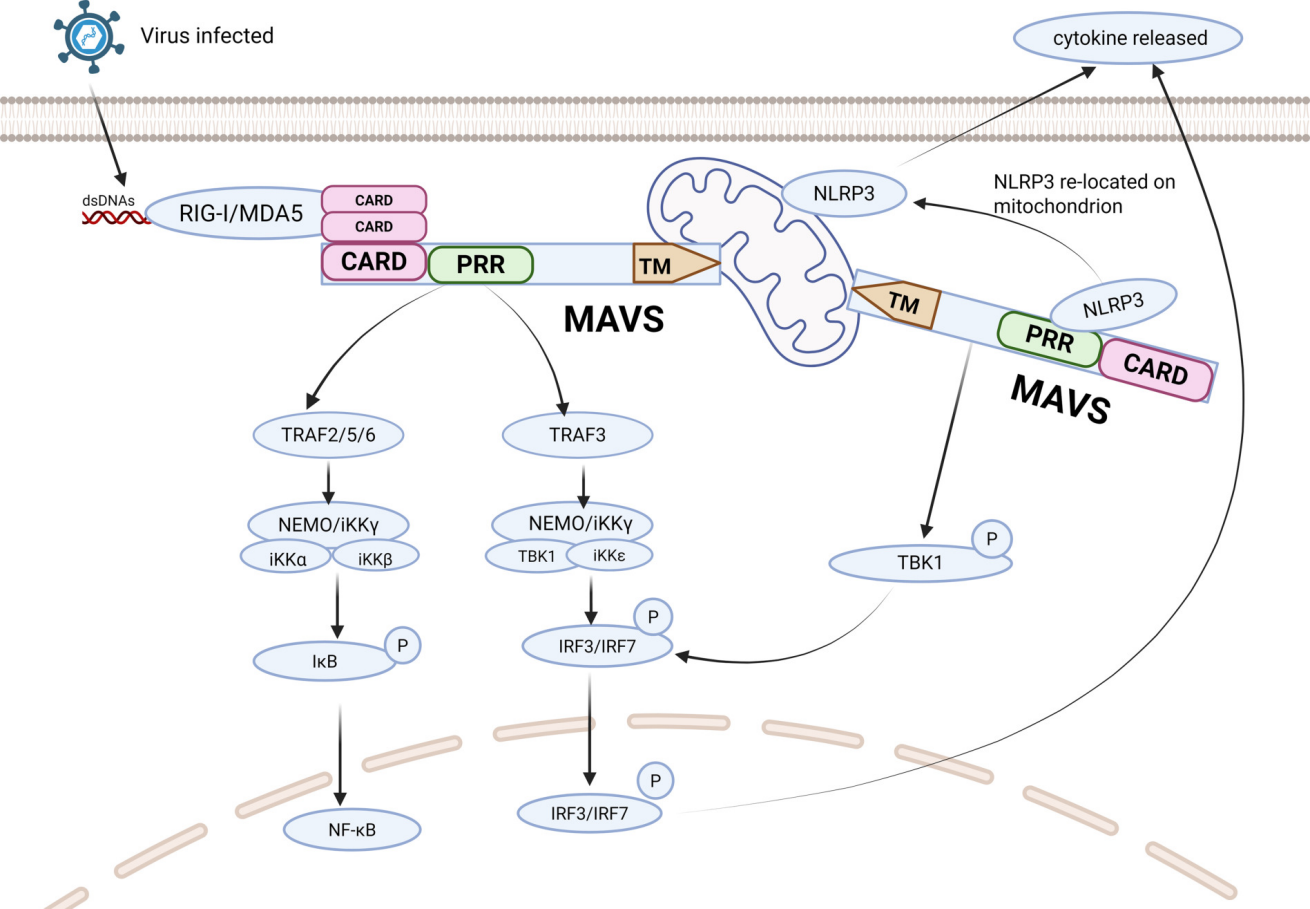
Represented illustration of MAVS antiviral signal simulation pathway. Interactions between CARD domains enable RIG-I and MDA5 activated by viruses to interact with MAVS. Upon PRR stimulation, MAVS triggers downstream signaling cascades by recruiting TRAF. MAVS activates two cytosolic protein kinase complexes, leading to the production of immune factors. The TBK1 complex phosphorylates IRF-3/7, promoting the transcription of IFN genes. Simultaneously, the IKK complex activates NF-kB, resulting in the production of proinflammatory cytokines. NLRP3 binds to MAVS to promote the production of IL-1β through its PYD in PRR. CARD, caspase activation and recruitment domain; PRR, proline-rich region; TM, transmembrane domain; RIG-Ⅰ, retinoic acid-inducible gene I; MDA5, melanoma differentiation-associated gene 5; NLRP3, NOD-like receptor protein 3; TRAF, tumor necrosis factor receptor associated factor. Created with Biorender.com.

Among the intensive studies, it has been found that MAVS can also bind to NOD-like receptor thermal protein domain protein 3 (NLRP3) that is recruited to the mitochondria to promote the production of IL-1β. It was also unveiled that the N-terminal pyrin domain (PYD) of NLRP3 is the key sequence mediating the NLRP3-MAVS interaction and localizing NLRP3 to the mitochondria (3) (Figure 2).

At the organ level, MAVS is substantially expressed in the heart, skeletal muscles, liver, and placenta. At the cellular level, it is distributed in various immune cells like macrophages, dendritic cells, monocytes, and other types of cells like epithelial cells and hepatocytes. At the subcellular level, it is located on the outer mitochondrial membrane, peroxisomes, and mitochondria-associated endoplasmic reticulum membranes (MAM). MAVS located on peroxisomes directly participates in a faster innate immune response which is independent of mitochondria to protect the body from pathogen invasion (7).

Although the function of MAVS in antiviral immune responses has been extensively studied, the significant role it plays in inflammatory response, apoptosis, and mitochondrial homeostasis has gradually drawn attention to the role of MAVS in cardiovascular diseases. Research indicates that abnormal activation or dysregulation of MAVS may be associated with various cardiovascular diseases, including myocarditis, heart failure, and myocardial infarction. Therefore, understanding the molecular structure and biological function of MAVS not only aids in revealing its role in innate immunity but also provides a foundation for exploring its potential role in cardiovascular diseases.

## 3 Mechanism of MAVS in cardiovascular diseases

### 3.1 MAVS in viral myocarditis

Viral myocarditis is an inflammatory destruction of the myocardium caused by cardiotropic viral infections such as Coxsackie B virus, human herpesvirus, etc., and is a common cause of dilated cardiomyopathy and heart attack (8). MAVS is extremely important for the development of viral myocarditis. Serrano et al. found that in normal cardiomyocytes, MAVS can be spontaneously activated, subsequently expressing high baseline levels of IFN-β to prevent viral invasion, and the MAVS on MAM is the necessity for the high expression of IFN-β (9). Liu et al. discovered that in cardiomyocytes infected with Coxsackievirus B3 (CVB3), the expression of TRIM21 increases, which can interact with MAVS and catalyze the k27-linked polyubiquitination of MAVS, promoting the activation of IRF3 and the transduction of IFN-β signaling to mitigate virus-induced cardiac damage (10). Additionally, research carried by Bazzone has demonstrated that under the stimulation of encephalomyocarditis virus (EMCV) RNA, A Disintegrin and Metalloproteinase domain 9 (ADAM9) (a metalloproteinase) activates downstream MAVS by binding to MDA5 and promoting its oligomerization, thereby enhancing antiviral signaling during viral infection (11). Fang et al. uncovered that TRIM18 exerts exacerbation of viral myocarditis by recruiting protein phosphatase 1A (PPM1A) to dephosphorylate TANK-binding kinase 1 (TBK1), preventing TBK1 from interacting with its MAVS and STING, thereby inhibiting antiviral signaling transduction, and knockdown TRIM18 can reduce less cardiac inflammation (12).

### 3.2 MAVS in heart failure

Heart failure (HF) is a series of clinical symptoms caused by the heart's inability to meet the body's metabolic needs due to cardiac dysfunction. In the late stages of heart failure, irreversible ventricular dilation and ventricular remodeling always occur, which caused severe outcomes (13). Research by Wang et al. underlined that MAVS is involved in the main mechanism of HF occurrence by affecting lipid metabolism and mitochondrial function. The expressions of MAVS show entirely converse in LPS-treated and Angiotensin Ⅱ (Ang Ⅱ)-treated hypertrophic hearts, which shows reduced expression level of MAVS in LPS-treated mice and enhanced MAVS expression in Ang Ⅱ-treated mice respectively. Hearts in MAVS^−/−^ mice showed downregulated levels of several fatty acids. Also, Phosphatidylcholines (PC) were found to be reduced, while the levels of PC catabolites increased, indicating that MAVS deletion might increase cell membrane decomposition or decrease cellular turnover, thereby limiting cardiomyocyte growth. Genes involved in fatty acid metabolism were downregulated and fat accumulation increased in MAVS^−/−^ mice, indicating that MAVS deficiency contributes to reduced energy generation in the heart. Moreover, the mitochondria of MAVS^−/−^ cardiomyocytes contained disrupted and disappeared ridges with a decrease in mitochondrial membrane potential (MMP) and mitochondrial autophagosomes, and mitophagy marker proteins were upregulated. The levels of byproducts in response to oxidative stress were found to be upgraded, suggesting that mitochondrial damage exacerbates by inducing oxidative stress and MAVS loss can impair the mitophagy flux. Together, MAVS loss shows mitochondrial damage by inducing mitochondrial ROS generation and abnormal mitophagy (14).

### 3.3 MAVS in myocardial infarction and MIRI

Myocardial Infarction (MI) refers to the pathophysiological process where local myocardial ischemia and hypoxia occur due to coronary artery blockage, leading to necrosis. It has a high incidence and mortality rate globally (15). Numerous studies have shown that inflammasome activation and autophagy play important roles in the pathogenesis of acute myocardial infarction (15, 16). Tax1 binding protein 1 (TAX1BP1) (a selective macro/autophagy receptor) participates in the termination of pro-inflammatory signaling and plays a significant role in host defense against pathogens and regulation of the innate immune system (17). Xu et al. found that TAX1BP1 inhibits the interaction between NLRP3 and MAVS by suppressing the localization of NLRP3 to mitochondria, thereby eliminating acute myocardial infarction-induced NLRP3 inflammasome activation and related mitochondrial dysfunction, ultimately alleviating myocardial infarction and cardiac dysfunction. The study also mentioned that RNF34, after being recruited by MAVS, interacts with TAX1BP1 to promote K27-linked MAVS polyubiquitination, thereby facilitating the autophagic degradation of MAVS. Silencing RNF34 can reduce hypoxia-induced MAVS aggregation in mitochondria, NLRP3 inflammasome activation, and associated mitochondrial membrane potential loss (18).

**Figure 3:**
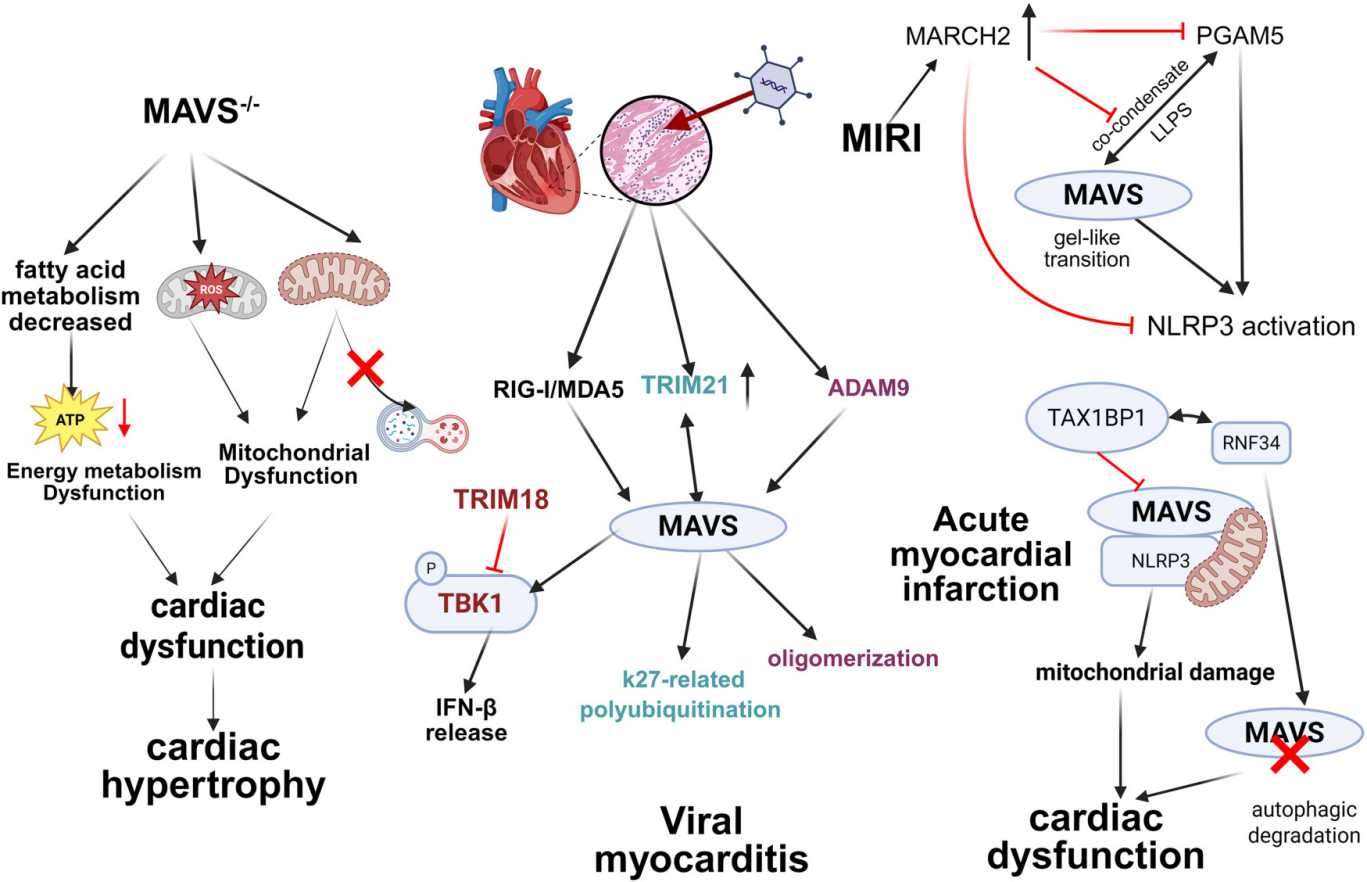
Main mechanisms of MAVS in cardiovascular diseases. The role of MAVS in viral myocarditis, MIRI, acute myocardial infarction, and HF. The knock down of MAVS or abnormal activation causes different pathological changes such as lipid metabolism disturbing, inflammation and mitochondrial damage, leading to cardiac dysfunction and aggravating HF. When viruses are infected, RIG-I and MDA5 interact with MAVS and activate the antiviral signal pathway; the expression of TRIM21 is upgraded and can cause k27-linked polyubiquitination of MAVS; ADAM9 leads to MAVS oligomerization. In AMI, TAX1BP1 interacts with RNF34, interrupting the autophagic degradation of MAVS, and inhibits the interacting between MAVS and NLRP3, leading to mitochondrial damage and exacerbate cardiac dysfunction. Created with Biorender.com.

Myocardial ischemia-reperfusion injury (MIRI) refers to the situation where, after ischemia occurs in the myocardium due to coronary artery constriction, although blood perfusion is restored through percutaneous coronary intervention (PCI) or other methods, the structure and function of myocardial cells in ischemia further deteriorate during this process, manifested as arrhythmia, decreased cardiac function, apoptosis or necrosis of myocardial cells. Many complex pathophysiological processes, such as oxidative stress, Ca^2+^ overload, inflammatory response, cell death, and autophagy, are involved in mediating MIRI (19–21). Recently, a research has unfolded that the membrane-associated RING finger protein 2 (MARCH2), an E3 ubiquitin ligase, directly interacts with phosphoglycerate mutase 5 (PGAM5) and facilitates K48-linked polyubiquitination and proteasomal degradation of MAVS, thereby inhibiting the activation of the NLRP3 inflammasome and reducing MIRI in cardiomyocytes (22) (Figure 3).

## 4 Regulation of MAVS activity

### 4.1 Post-translational modifications (PTMs) in MAVS and cardiovascular diseases

Post-translational modifications (PTMs) entail the conjugation of various biochemical functional groups to proteins after translation, which alters the chemical properties of amino acids or induces structural changes, thereby enhancing protein functions. The most crucial step in the activation process of MAVS is the CARD-related PTMs. Therefore, extensive research has been done on MAVS-related PTMs. While in cardiovascular diseases, though the PTMs mentioned above have been discussed thoroughly in other virus infected diseases, there's still a lack of evidence of the direct connections between PTMs of MAVS and cardiovascular diseases. Therefore, the method to manipulate PTMs of MAVS could be a new direction for cardiovascular disease treatments. Here is the summary of PTMs that regulate MAVS or participate in cardiovascular diseases.

#### 4.1.1 Ubiquitination

Ubiquitination is the process by which ubiquitin molecules, under the action of a series of special enzymes, categorize intracellular proteins, select target protein molecules, and perform specific modifications on the target proteins. Currently, the ubiquitination of MAVS mainly focuses on Lysine 27 (K27), K63, and K48-linked ubiquitination. Briefly speaking, MAVS can be K63-linked polyubiquitinated to enhance its downstream signaling activity by promoting the recruitment of TRAFs and activation of the TBK1/IKKε complex, whereas K48-linked polyubiquitination facilitates its proteasomal degradation and suppresses its activity. While in K27-linked ubiquitination, its role in regulating MAVS and their related signaling pathways is complex. For instance, IFN-induced BST2 recruits MARCH8 to catalyze the K27-linked ubiquitination of MAVS for autophagic degradation, hence inhibiting type I interferon signaling (23). RNF34 facilitates the autophagic degradation of MAVS by targeting its K27-linked ubiquitination (24). But UBL7 enhances antiviral innate immunity by promoting K27-linked polyubiquitination of MAVS (Table 1).

**Table 1:** Ubiquitination regulation related proteins of MAVS.

MAVS Regulatory mechanism	Regulatory factors	Relevant references
K27 linked Ubiquitination	TRIM21, UBL7, MARCH8	(55–57)
K63 linked Ubiquitination	TRIM31, USP18, N4DP3	(58–60)
K48 linked Ubiquitination	TRIM28, SMURF1, SMURF2, TRIM25, OTUD4, TRIM44, MARCH5, RNF5, RNF146	(61–69)

In atherosclerosis, persistent K63-linked signaling may promote macrophage activation and foam cell formation through enhancing NLRP3 inflammasome activation (25). Other ubiquitin-regulated proteins (e.g., YAP, SR-A1, SR-B1) undergo K63 ubiquitination that modulates lipid uptake and foam cell formation (26).

#### 4.1.2 Methylation

Methylation is a key modification in proteins and nucleic acids (27), among which arginine methylation mediated by arginine methyltransferase (PRMT) is an important PTM that can regulate various cellular processes (28). Research has demonstrated that arginine monomethylation serves as a negative regulatory mechanism to MAVS-mediated antiviral response and is involved in maintaining immune homeostasis. Wang et al. demonstrated that PRMT7 suppresses the oligomerization of MAVS and its downstream interferon signaling (29). Bai et al. discovered that PRMT9 can catalyze arginine methylation of MAVS at the Arg41 and Arg43 sites to inhibit MAVS aggregation and auto-activation. After viral infection, PRMT9 dissociates from MAVS on the mitochondria, allowing MAVS aggregation and activation (30). While PRMT5 has not yet been directly shown to methylate MAVS, it symmetrically dimethylates various immune-related proteins, raising the possibility of MAVS as a future substrate.

When it comes to cardiovascular diseases, PRMTs dysregulation precipitates endothelial dysfunction, resulting in increased permeability, aberrant vasodilation, and inflammatory response, thereby culminating in atherosclerosis (31). Besides, PRMTs actively participate in blood pressure regulation by influencing vascular tone and endothelial function (31, 32).

#### 4.1.3 Acetylation

Acetylation refers to the process in which an acetyl group from acetyl coenzyme A (acetyl-CoA) is transferred to an amino acid residue of a protein under the action of acetyltransferase. Lysine acetylation modification in proteins also regulates various properties of proteins, including DNA-protein interactions, subcellular localization, transcriptional activity, protein stability, etc. Recent studies have demonstrated that sirtuin3 (SIRT3) and SIRT5 play important regulatory roles as targets for cardiovascular diseases (33, 34). Other studies have also discovered that SIRT3 can interact with MAVS to catalyze the deacetylation of MAVS at lysine residue 7 (K7), promoting MAVS aggregation, leading to increased MAVS activation and enhanced type I IFN signaling. SIRT3 knockout can cause the increasing viral susceptibility, while knocking out SIRT5 may counteract the action caused by SIRT3 knockout. Therefore, this study hypothesizes that SIRT3 may positively regulate antiviral immunity through MAVS, while SIRT5 may act as its antagonist to coordinate antiviral innate immunity (35) (Table 2).

**Table 2:** PTMs of MAVS and of cardiovascular diseases.

PTM type	MAVS regulatory effect	Potential cardiovascular relevance
Ubiquitination	K27 and K63-linked promote signaling, K27 and K48-linked induce degradation	Viral myocarditis, promote macrophage activation and foam cell formation through enhancing NLRP3 activation in AS
Arginine methylation	Inhibits oligomerization, dampens IFN-I signaling	Immune overactivation control, protection from tissue damage
Acetylation	May regulate mitochondrial localization/function	Energy stress adaptation, ischemia-related cardio-protection

### 4.2 Other regulatory pathways for MAVS

In addition to classical interactors and PTMs, MAVS activity is modulated by various microRNAs and mitochondria-associated proteins, many of which also play important roles in cardiovascular diseases. Several miRNAs have been identified as regulators of MAVS expression or functions. miR-125a directly targets MAVS and suppresses its expression, thereby attenuating type I interferon responses. miR-33/33* inhibits AMPK signaling and indirectly reduces MAVS aggregation, impairing mitophagy and mitochondrial homeostasis—mechanisms closely linked to atherosclerosis. The miR-302/367 cluster, known to promote myocardial regeneration post-myocardial infarction, can inhibit MAVS activation by downregulating SLC25A12, a mitochondrial carrier essential for RLR signaling.

Moreover, several mitochondrial membrane proteins directly interact with MAVS to regulate its activity. Tom70, a mitochondrial import receptor, binds MAVS and facilitates IRF3-dependent antiviral signaling (36, 37). Downregulation of Tom70 exacerbates post-MI injury, increases ROS production, and promotes maladaptive cardiac hypertrophy (38). Mitofusin 2 (MFN2) interacts with the C-terminus of MAVS to suppress RIG-I/MDA5 signaling (39). MFN2 also improves cardiac oxidative balance and mitochondrial ATP production (40). Optic Atrophy 1 (OPA1) is associated with MAVS to maintain mitochondrial structure and function. Knockdown of OPA1 mimics MAVS deficiency and accelerates cellular senescence, while reintroduction of either protein restores mitochondrial homeostasis in stem cells (41). Toll-interacting protein (TOLLIP), a negative regulator of RLR signaling and a modulator of autophagy, enhances the interaction between MAVS and the SUMO protease SENP1, promoting deSUMOylation and reducing MAVS aggregation (42). TOLLIP has been implicated in the regulation of several cardiovascular diseases, including atherosclerosis, cardiac hypertrophy, and myocardial infarction (43–45). TTLL12, identified as a MAVS-binding protein with tubulin tyrosine ligase and methyltransferase activity, can suppress MAVS-mediated type I IFN production through direct interaction (46). Though the role of TTLL12 in cardiovascular diseases hasn't been discovered, it has been proved its key role in epithelial cells polarization, influencing cilia formation in polarized renal epithelial cells and anti-tumor immunity (47, 48) (Table 3).

**Table 3:** Other regulatory pathways of MAVS.

Other Regulatory Pathways	For MAVS	Relevance with Cardiovascular Diseases
miR-125a	Suppress MAVS expression and attenuate type I interferon responses (−)	Protection against MIRI; (70) suppress endothelial cell proliferation and high glucose-induced VSMC proliferation and migration (71, 72)
miR-33	Reduce MAVS aggregation, impairing mitophagy and mitochondrial homeostasis (−)	Inhibit ABCA1/ABCG1-mediated cholesterol efflux of macrophages in AS (73)
miR-302b	Inhibit MAVS activation by downregulating SLC25A12 (−)	Promote cardiomyocyte proliferation and functional regeneration after MIRI (74)
OPA1	Knock down OPA1 causes mitochondrial structural and functional damage caused by MAVS deficiency, accelerating cellular aging (−)	Imbalanced OPA1 processing and mitochondrial fragmentation aggregate HF (75); promote mitochondrial fusion against DCM (76)
TOM70	Binds MAVS and facilitates IRF3-dependent antiviral signaling (+)	Attenuate post-MIRI and MIRI; (36, 77) protect cardiomyocytes from myocardial hypertrophy; (38)
MFN2	Interacts with the C-terminus of MAVS to suppress RIG-I/MDA5 signaling (−)	Improve cardiac oxidative balance and mitochondrial ATP production in HF, restore mitochondrial function in DCM; (78) alleviate drug-induced cardiotoxicity (79, 80)
TOLLIP	Enhance the interaction between SENP1 and MAVS, leading to deSUMOylation and less aggregation of MAVS (−)	Disrupting Lipophagy in AS (45); anti-hypertrophic effects (43); promoting inflammation and apoptosis in MI (44); attenuate the hypertrophic response of cardiomyocytes induced by IL-1β (81)
TTLL12	Interact with MAVS and inhibit I-IFN expression (−)	—

(+): Indicates activating MAVS-related antiviral signal pathway and promoting MAVS aggregation. (−): Indicates the inhibition of MAVS activation or mitochondrial dysfunction.

## 5 MAVS-targeted therapies in different diseases

Although MAVS-targeted therapies are still in the early stages of development, their potential applications are broad. Preliminary studies suggest that MAVS-targeted strategies could become an integral component of combination therapies for virus-related cancers and resistant malignancies (49–52). Furthermore, these approaches may be valuable in treating autoimmune and inflammatory conditions where MAVS signaling is dysregulated (53). As more clinical data accumulates, MAVS-targeted therapies are expected to emerge as novel strategies for managing complex immune and inflammatory diseases, which could open new avenues for MAVS-targeted immunotherapy, not only in virus-associated tumors but also in a range of inflammatory and autoimmune disorders.

Given the diverse and complex mechanism of the pathogenesis of cardiovascular diseases, when MAVS is absent, it will damage mitochondrial function, increase oxidative stress, and lead to the deterioration of the disease. As the research has shown, supplementing MAVS can alleviate functional damage of mitochondria. Therefore, using MAVS agonists for these diseases may have therapeutic effects. Also, MAVS is involved in the activation of the antiviral signaling pathway and inflammasome, so MAVS inhibitors can, to some extent, alleviate certain inflammatory diseases such as atherosclerosis. Various viruses have evolved immune evasion mechanisms to avoid the activation of MAVS, the treatments for specific viral infections should be adjusted instead of focusing on MAVS itself. The goal is to achieve precise immunomodulation and alleviate disease symptoms without compromising the host's antiviral defenses.

## 6 Conclusions and perspectives

In summary, MAVS, as a key mitochondria-associated receptor protein, plays an important role in innate immunity and the pathophysiological processes of various cardiovascular diseases. During viral infections, MAVS recruits its interacting proteins and downstream molecules, activates NF-κB and IRF signaling, mediates the production of IFN, and plays a crucial role in mitochondria-mediated antiviral innate immune responses. Moreover, MAVS also significantly impacts the inflammatory response of cardiomyocytes and various immune cells by activating the NLRP3 inflammasome, potentially having critical regulatory significance in the development of various cardiovascular diseases.

### 6.1 Regulation mechanism of MAVS

Various post-translational modifications are involved in regulating MAVS activity and the activation of its mediated signaling pathways; miRNA regulation of MAVS and various proteins can interact with MAVS to regulate its own activity or related pathways. Research on these pathways and the application of MAVS agonists and antagonists may provide new therapeutic strategies for future cardiovascular disease research.

### 6.2 The potential role of MAVS in cardiovascular diseases

MAVS shows an important role in cardiovascular disease models such as viral myocarditis, heart failure, MIRI, and myocardial infarction, suggesting that MAVS has application value as a diagnostic and therapeutic target for cardiovascular diseases.

### 6.3 Research gaps and challenges about MAVS

#### 6.3.1 Insufficient clinical research in cardiology

Most MAVS-related studies to date have focused on viral immunity, oncology, or autoimmune conditions. Despite accumulating preclinical evidence indicating that MAVS may influence cardiovascular homeostasis, direct clinical studies evaluating MAVS expression or activity in human cardiac tissues or patient cohorts are scarce. Particularly, there is a lack of:

• Clinical correlation between MAVS levels and cardiovascular disease severity or prognosis.
• Serum biomarker analyses for MAVS or related mitochondrial proteins.
• Integration of MAVS-related indices into existing cardiovascular risk models.

Furthermore, while surrogate inflammatory indicators such as the neutrophil-to-lymphocyte ratio (NLR) have been associated with mitochondrial stress and RIG-I–MAVS signaling (54), no direct clinical linkage between MAVS activation and NLR dynamics has been established.

#### 6.3.2 Incomplete mechanistic understanding of MAVS regulation in cardiovascular contexts

Although post-translational modifications (PTMs) such as ubiquitination, methylation, and acetylation of MAVS have been extensively studied in the context of viral infections, their direct roles in cardiovascular diseases remain poorly characterized. Key knowledge gaps include:

• The functional consequences of specific MAVS PTMs (e.g., K27-linked ubiquitination) in cardiomyocytes.
• Whether disease-specific stimuli (e.g., ischemia, oxidative stress, mechanical overload) selectively influences MAVS activity.
• The organellar-specific roles of MAVS (e.g., mitochondrial outer membrane vs. MAMs or peroxisomes) in metabolic reprogramming of the failing heart.

Future studies employing cardiac-specific MAVS mutants and PTM-deficient models are needed to dissect these mechanisms.

#### 6.3.3 Translational challenges in MAVS-targeted therapeutic developments

While MAVS agonists or antagonists have shown promise in regulating immune responses, their application in cardiovascular diseases is still hypothetical. Major barriers include:

• Lack of MAVS-selective modulators with proven efficacy and safety in cardiovascular settings.
• Uncertainty regarding the therapeutic window: MAVS activation may enhance antiviral protection but also exacerbate inflammation.
• Off-target effects due to MAVS expression in non-cardiac tissues (e.g., immune cells, liver).

Targeted delivery strategies, such as tissue-specific nanoparticles or cardiac-tropic gene therapy vectors, may be required to address these challenges.

## References

[1] Liu D Qin H Gao Y Sun M Wang M. Cardiovascular disease: mitochondrial dynamics and mitophagy crosstalk mechanisms with novel programmed cell death and macrophage polarisation. Pharmacol Res. (2024) 206:107258. 10.1016/j.phrs.2024.107258 38909638

[2] Ning X Wang Y Jing M Sha M Lv M Gao P Apoptotic caspases suppress type I interferon production via the cleavage of cGAS, MAVS, and IRF3. Mol Cell. (2019) 74(1):19–31.e7. 10.1016/j.molcel.2019.02.013 30878284

[3] Subramanian N Natarajan K Clatworthy MR Wang Z Germain RN. The adaptor MAVS promotes NLRP3 mitochondrial localization and inflammasome activation. Cell. (2013) 153(2):348–61. 10.1016/j.cell.2013.02.054 23582325 PMC3632354

[4] Seth RB Sun L Ea CK Chen ZJ. Identification and characterization of MAVS, a mitochondrial antiviral signaling protein that activates NF-kappaB and IRF 3. Cell. (2005) 122(5):669–82. 10.1016/j.cell.2005.08.012 16125763

[5] Kawai T Takahashi K Sato S Coban C Kumar H Kato H IPS-1, an adaptor triggering RIG-I- and Mda5-mediated type I interferon induction. Nat Immunol. (2005) 6(10):981–8. 10.1038/ni1243 16127453

[6] Hou F Sun L Zheng H Skaug B Jiang QX Chen ZJ. MAVS forms functional prion-like aggregates to activate and propagate antiviral innate immune response. Cell. (2011) 146(3):448–61. 10.1016/j.cell.2011.06.041 21782231 PMC3179916

[7] Dixit E Boulant S Zhang Y Lee AS Odendall C Shum B Peroxisomes are signaling platforms for antiviral innate immunity. Cell. (2010) 141(4):668–81. 10.1016/j.cell.2010.04.018 20451243 PMC3670185

[8] Sagar S Liu PP Cooper LT Jr. Myocarditis. Lancet. (2012) 379(9817):738–47. 10.1016/S0140-6736(11)60648-X 22185868 PMC5814111

[9] Rivera-Serrano EE DeAngelis N Sherry B. Spontaneous activation of a MAVS-dependent antiviral signaling pathway determines high basal interferon-β expression in cardiac myocytes. J Mol Cell Cardiol. (2017) 111:102–13. 10.1016/j.yjmcc.2017.08.008 28822807 PMC5600710

[10] Liu H Li M Song Y Xu W. TRIM21 restricts coxsackievirus B3 replication, cardiac and pancreatic injury via interacting with MAVS and positively regulating IRF3-mediated type-I interferon production. Front Immunol. (2018) 9:2479. 10.3389/fimmu.2018.02479 30410495 PMC6209670

[11] Bazzone LE Zhu J King M Liu G Guo Z MacKay CR ADAM9 promotes type I interferon-mediated innate immunity during encephalomyocarditis virus infection. Nat Commun. (2024) 15(1):4153. 10.1038/s41467-024-48524-6 38755212 PMC11098812

[12] Fang M Zhang A Du Y Lu W Wang J Minze LJ TRIM18 is a critical regulator of viral myocarditis and organ inflammation. J Biomed Sci. (2022) 29(1):55. 10.1186/s12929-022-00840-z 35909127 PMC9339186

[13] Ziaeian B Fonarow GC. Epidemiology and aetiology of heart failure. Nat Rev Cardiol. (2016) 13(6):368–78. 10.1038/nrcardio.2016.25 26935038 PMC4868779

[14] Wang Q Sun Z Cao S Lin X Wu M Li Y Reduced immunity regulator MAVS contributes to non-hypertrophic cardiac dysfunction by disturbing energy metabolism and mitochondrial homeostasis. Front Immunol. (2022) 13:919038. 10.3389/fimmu.2022.919038 35844503 PMC9283757

[15] Abbate A Toldo S Marchetti C Kron J Van Tassell BW Dinarello CA. Interleukin-1 and the inflammasome as therapeutic targets in cardiovascular disease. Circ Res. (2020) 126(9):1260–80. 10.1161/CIRCRESAHA.120.315937 32324502 PMC8760628

[16] Bravo-San Pedro JM Kroemer G Galluzzi L. Autophagy and mitophagy in cardiovascular disease. Circ Res. (2017) 120(11):1812–24. 10.1161/CIRCRESAHA.117.311082 28546358

[17] White J Suklabaidya S Vo MT Choi YB Harhaj EW. Multifaceted roles of TAX1BP1 in autophagy. Autophagy. (2023) 19(1):44–53. 10.1080/15548627.2022.2070331 35470757 PMC9809930

[18] Xu H Yu W Sun S Li C Ren J Zhang Y. TAX1BP1 protects against myocardial infarction-associated cardiac anomalies through inhibition of inflammasomes in a RNF34/MAVS/NLRP3-dependent manner. Sci Bull. (2021) 66(16):1669–83. 10.1016/j.scib.2021.01.030 36654301

[19] Li J Zhang J Zhong Y Xie D Han H Zhang Z TRPC6 regulates necroptosis in myocardial ischemia/reperfusion injury via Ca(2+)/CaMKII signaling pathway. Cell Signal. (2024) 122:111344. 10.1016/j.cellsig.2024.111344 39134250

[20] Yang Y Zhang Y Yang J Zhang M Tian T Jiang Y Interdependent nuclear co-trafficking of ASPP1 and p53 aggravates cardiac ischemia/reperfusion injury. Circ Res. (2023) 132(2):208–22. 10.1161/CIRCRESAHA.122.321153 36656967 PMC9855749

[21] Xing Y Sui Z Liu Y Wang MM Wei X Lu Q Blunting TRPML1 channels protects myocardial ischemia/reperfusion injury by restoring impaired cardiomyocyte autophagy. Basic Res Cardiol. (2022) 117(1):20. 10.1007/s00395-022-00930-x 35389129

[22] Liu S Bi Y Han T Li YE Wang Q Wu NN The E3 ubiquitin ligase MARCH2 protects against myocardial ischemia-reperfusion injury through inhibiting pyroptosis via negative regulation of PGAM5/MAVS/NLRP3 axis. Cell Discov. (2024) 10(1):24. 10.1038/s41421-023-00622-3 38409220 PMC10897310

[23] Jin S Cui J. BST2 inhibits type I IFN (interferon) signaling by accelerating MAVS degradation through CALCOCO2-directed autophagy. Autophagy. (2018) 14(1):171–2. 10.1080/15548627.2017.1393590 29165031 PMC5846508

[24] He X Zhu Y Zhang Y Geng Y Gong J Geng J RNF34 functions in immunity and selective mitophagy by targeting MAVS for autophagic degradation. EMBO J. (2019) 38(14):e100978. 10.15252/embj.2018100978 31304625 PMC6627233

[25] Zhou Z Zhu X Yin R Liu T Yang S Zhou L K63 ubiquitin chains target NLRP3 inflammasome for autophagic degradation in ox-LDL-stimulated THP-1 macrophages. Aging. (2020) 12(2):1747–59. 10.18632/aging.102710 32003754 PMC7053591

[26] Wang B Tang X Yao L Wang Y Chen Z Li M Disruption of USP9X in macrophages promotes foam cell formation and atherosclerosis. J Clin Invest. (2022) 132(10):e154217. 10.1172/JCI154217 35389885 PMC9106359

[27] Clarke S. Protein methylation. Curr Opin Cell Biol. (1993) 5(6):977–83. 10.1016/0955-0674(93)90080-A 8129951

[28] Zheng K Chen S Ren Z Wang Y. Protein arginine methylation in viral infection and antiviral immunity. Int J Biol Sci. (2023) 19(16):5292–318. 10.7150/ijbs.89498 37928266 PMC10620831

[29] Zhu J Li X Cai X Zha H Zhou Z Sun X Arginine monomethylation by PRMT7 controls MAVS-mediated antiviral innate immunity. Mol Cell. (2021) 81(15):3171–86.e8. 10.1016/j.molcel.2021.06.004 34171297

[30] Bai X Sui C Liu F Chen T Zhang L Zheng Y The protein arginine methyltransferase PRMT9 attenuates MAVS activation through arginine methylation. Nat Commun. (2022) 13(1):5016. 10.1038/s41467-022-32628-y 36028484 PMC9418238

[31] Tan B Liu Q Yang L Yang Y Liu D Liu L Low expression of PRMT5 in peripheral blood may serve as a potential independent risk factor in assessments of the risk of stable CAD and AMI. BMC Cardiovasc Disord. (2019) 19(1):31. 10.1186/s12872-019-1008-4 30704408 PMC6357489

[32] Melikian N Seddon MD Casadei B Chowienczyk PJ Shah AM. Neuronal nitric oxide synthase and human vascular regulation. Trends Cardiovasc Med. (2009) 19(8):256–62. 10.1016/j.tcm.2010.02.007 20447567 PMC2984617

[33] Tang WH Tong W Shrestha K Wang Z Levison BS Delfraino B Differential effects of arginine methylation on diastolic dysfunction and disease progression in patients with chronic systolic heart failure. Eur Heart J. (2008) 29(20):2506–13. 10.1093/eurheartj/ehn360 18687662 PMC2567021

[34] Jin L Geng L Ying L Shu L Ye K Yang R FGF21-Sirtuin 3 axis confers the protective effects of exercise against diabetic cardiomyopathy by governing mitochondrial integrity. Circulation. (2022) 146(20):1537–57. 10.1161/CIRCULATIONAHA.122.059631 36134579

[35] Liu X Zhu C Jia S Deng H Tang J Sun X Dual modifying of MAVS at lysine 7 by SIRT3-catalyzed deacetylation and SIRT5-catalyzed desuccinylation orchestrates antiviral innate immunity. Proc Natl Acad Sci U S A. (2024) 121(17):e2314201121. 10.1073/pnas.2314201121 38635631 PMC11047105

[36] Pei HF Hou JN Wei FP Xue Q Zhang F Peng CF Melatonin attenuates postmyocardial infarction injury via increasing Tom70 expression. J Pineal Res. (2017) 62(1):e12371. 10.1111/jpi.12371 27706848

[37] Liu XY Wei B Shi HX Shan YF Wang C. Tom70 mediates activation of interferon regulatory factor 3 on mitochondria. Cell Res. (2010) 20(9):994–1011. 10.1038/cr.2010.103 20628368

[38] Li J Qi M Li C Shi D Zhang D Xie D Tom70 serves as a molecular switch to determine pathological cardiac hypertrophy. Cell Res. (2014) 24(8):977–93. 10.1038/cr.2014.94 25022898 PMC4123302

[39] Yasukawa K Oshiumi H Takeda M Ishihara N Yanagi Y Seya T Mitofusin 2 inhibits mitochondrial antiviral signaling. Sci Signal. (2009) 2(84):ra47. 10.1126/scisignal.2000287 19690333

[40] Chung E Joiner HE Skelton T Looten KD Manczak M Reddy PH. Maternal exercise upregulates mitochondrial gene expression and increases enzyme activity of fetal mouse hearts. Physiol Rep. (2017) 5(5):e13184. 10.14814/phy2.13184 28292876 PMC5350185

[41] Wang C Yang K Liu X Wang S Song M Belmonte JCI MAVS antagonizes human stem cell senescence as a mitochondrial stabilizer. Research. (2023) 6:0192. 10.34133/research.0192 37521327 PMC10374246

[42] Hou J Zheng S Zhang X Zhuang M Zhao X Deng J IDR-driven TOLLIP condensates antagonize the innate antiviral immunity by promoting the deSUMOylation of MAVS. Cell Rep. (2025) 44(3):115348. 10.1016/j.celrep.2025.115348 40022729

[43] Liu Y Jiang XL Liu Y Jiang DS Zhang Y Zhang R Toll-interacting protein (tollip) negatively regulates pressure overload-induced ventricular hypertrophy in mice. Cardiovasc Res. (2014) 101(1):87–96. 10.1093/cvr/cvt232 24285748 PMC3968303

[44] Wan N Liu X Zhang XJ Zhao Y Hu G Wan F Toll-interacting protein contributes to mortality following myocardial infarction through promoting inflammation and apoptosis. Br J Pharmacol. (2015) 172(13):3383–96. 10.1111/bph.13130 25765712 PMC4500373

[45] Chen K Yuan R Zhang Y Geng S Li L. Tollip deficiency alters atherosclerosis and steatosis by disrupting lipophagy. J Am Heart Assoc. (2017) 6(4):e004078. 10.1161/JAHA.116.004078 28396568 PMC5532987

[46] Ju LG Zhu Y Lei PJ Yan D Zhu K Wang X TTLL12 inhibits the activation of cellular antiviral signaling through interaction with VISA/MAVS. J Immunol. (2017) 198(3):1274–84. 10.4049/jimmunol.1601194 28011935

[47] Chen DW Cheng YK Li PS Wu XJ Huang XX Long JH Tumor-intrinsic TTLL12 drives resistance to cancer immunotherapy via modulating myeloid-derived suppressor cells. J Immunother Cancer. (2025) 13(6):e010873. 10.1136/jitc-2024-010873 40461158 PMC12142039

[48] Ceglowski J Hoffman HK Neumann AJ Hoff KJ McCurdy BL Moore JK TTLL12 is required for primary ciliary axoneme formation in polarized epithelial cells. EMBO Rep. (2024) 25(1):198–227. 10.1038/s44319-023-00005-5 38177908 PMC10883266

[49] Dultz G Shimakami T Schneider M Murai K Yamane D Marion A Extended interaction networks with HCV protease NS3-4A substrates explain the lack of adaptive capability against protease inhibitors. J Biol Chem. (2020) 295(40):13862–74. 10.1074/jbc.RA120.013898 32747444 PMC7535904

[50] Zhou L He R Fang P Li M Yu H Wang Q Hepatitis B virus rigs the cellular metabolome to avoid innate immune recognition. Nat Commun. (2021) 12(1):98. 10.1038/s41467-020-20316-8 33397935 PMC7782485

[51] Xiao D Liu D Wen Z Huang X Zeng C Zhou Z Interaction between susceptibility loci in MAVS and TRAF3 genes, and high-risk HPV infection on the risk of cervical precancerous lesions in Chinese population. Cancer Prev Res. (2019) 12(1):57–66. 10.1158/1940-6207.CAPR-18-0177 30463990

[52] Zitvogel L Galluzzi L Kepp O Smyth MJ Kroemer G. Type I interferons in anticancer immunity. Nat Rev Immunol. (2015) 15(7):405–14. 10.1038/nri3845 26027717

[53] Pothlichet J Niewold TB Vitour D Solhonne B Crow MK Si-Tahar M. A loss-of-function variant of the antiviral molecule MAVS is associated with a subset of systemic lupus patients. EMBO Mol Med. (2011) 3(3):142–52. 10.1002/emmm.201000120 21268286 PMC3395111

[54] Ranhbar A Sohrabi B Hajizadeh R Shoar MK Kavandi H Ghodratizadeh S Neutrophil-to-lymphocyte ratio and outpatient management of low-risk acute pulmonary embolism. Heart Mind. (2022) 6(3):183–6. 10.4103/hm.hm_20_21

[55] Xue B Li H Guo M Wang J Xu Y Zou X TRIM21 promotes innate immune response to RNA viral infection through Lys27-linked polyubiquitination of MAVS. J Virol. (2018) 92(14):10–128. 10.1128/JVI.00321-18 PMC6026736 29743353

[56] Jiang W Li X Xu H Gu X Li S Zhu L UBL7 enhances antiviral innate immunity by promoting Lys27-linked polyubiquitination of MAVS. Cell Rep. (2023) 42(3):112272. 10.1016/j.celrep.2023.112272 36943869

[57] Wang P Sun Y Xu T. USP13 cooperates with MARCH8 to inhibit antiviral signaling by targeting MAVS for autophagic degradation in teleost. J Immunol. (2024) 212(5):801–12. 10.4049/jimmunol.2300493 38214605

[58] Liu B Zhang M Chu H Zhang H Wu H Song G The ubiquitin E3 ligase TRIM31 promotes aggregation and activation of the signaling adaptor MAVS through Lys63-linked polyubiquitination. Nat Immunol. (2017) 18(2):214–24. 10.1038/ni.3641 27992402

[59] Wang C Ling T Zhong N Xu LG. N4BP3 regulates RIG-I-like receptor antiviral signaling positively by targeting mitochondrial antiviral signaling protein. Front Microbiol. (2021) 12:770600. 10.3389/fmicb.2021.770600 34880843 PMC8646042

[60] Hou J Han L Zhao Z Liu H Zhang L Ma C USP18 positively regulates innate antiviral immunity by promoting K63-linked polyubiquitination of MAVS. Nat Commun. (2021) 12(1):2970. 10.1038/s41467-021-23219-4 34016972 PMC8137702

[61] Chen YY Ran XH Ni RZ Mu D. TRIM28 negatively regulates the RLR signaling pathway by targeting MAVS for degradation via K48-linked polyubiquitination. J Biol Chem. (2023) 299(5):104660. 10.1016/j.jbc.2023.104660 37119745 PMC10165269

[62] Castanier C Zemirli N Portier A Garcin D Bidère N Vazquez A MAVS ubiquitination by the E3 ligase TRIM25 and degradation by the proteasome is involved in type I interferon production after activation of the antiviral RIG-I-like receptors. BMC Biol. (2012) 10:44. 10.1186/1741-7007-10-44 22626058 PMC3372421

[63] Pan Y Li R Meng JL Mao HT Zhang Y Zhang J. Smurf2 negatively modulates RIG-I-dependent antiviral response by targeting VISA/MAVS for ubiquitination and degradation. J Immunol. (2014) 192(10):4758–64. 10.4049/jimmunol.1302632 24729608

[64] Liuyu T Yu K Ye L Zhang Z Zhang M Ren Y Induction of OTUD4 by viral infection promotes antiviral responses through deubiquitinating and stabilizing MAVS. Cell Res. (2019) 29(1):67–79. 10.1038/s41422-018-0107-6 30410068 PMC6318273

[65] Park YJ Oanh NTK Heo J Kim SG Lee HS Lee H Dual targeting of RIG-I and MAVS by MARCH5 mitochondria ubiquitin ligase in innate immunity. Cell Signal. (2020) 67:109520. 10.1016/j.cellsig.2019.109520 31881323

[66] Zhong B Zhang Y Tan B Liu TT Wang YY Shu HB. The E3 ubiquitin ligase RNF5 targets virus-induced signaling adaptor for ubiquitination and degradation. J Immunol. (2010) 184(11):6249–55. 10.4049/jimmunol.0903748 20483786

[67] Ahlén G Derk E Weiland M Jiao J Rahbin N Aleman S Cleavage of the IPS-1/Cardif/MAVS/VISA does not inhibit T cell-mediated elimination of hepatitis C virus non-structural 3/4A-expressing hepatocytes. Gut. (2009) 58(4):560–9. 10.1136/gut.2007.147264 18689426 PMC2648966

[68] Zhang L Liu J Qian L Feng Q Wang X Yuan Y Induction of OTUD1 by RNA viruses potently inhibits innate immune responses by promoting degradation of the MAVS/TRAF3/TRAF6 signalosome. PLoS Pathog. (2018) 14(5):e1007067. 10.1371/journal.ppat.1007067 29734366 PMC5957451

[69] Liu W Ma Z Wu Y Yuan C Zhang Y Liang Z MST4 negatively regulates type I interferons production via targeting MAVS-mediated pathway. Cell Commun Signal. (2022) 20(1):103. 10.1186/s12964-022-00922-3 35820905 PMC9274187

[70] Díaz I Calderón-Sánchez E Toro RD Ávila-Médina J de Rojas-de Pedro ES Domínguez-Rodríguez A miR-125a, miR-139 and miR-324 contribute to urocortin protection against myocardial ischemia-reperfusion injury. Sci Rep. (2017) 7(1):8898. 10.1038/s41598-017-09198-x 28827743 PMC5566224

[71] Svensson D Gidlöf O Turczyńska KM Erlinge D Albinsson S Nilsson BO. Inhibition of microRNA-125a promotes human endothelial cell proliferation and viability through an antiapoptotic mechanism. J Vasc Res. (2014) 51(3):239–45. 10.1159/000365551 25116893

[72] Ye D Lou GH Li AC Dong FQ Chen GP Xu WW MicroRNA-125a-mediated regulation of the mevalonate signaling pathway contributes to high glucose-induced proliferation and migration of vascular smooth muscle cells. Mol Med Rep. (2020) 22(1):165–74. 10.3892/mmr.2020.11077 32319638 PMC7248521

[73] Mao M Lei H Liu Q Chen Y Zhao L Li Q Effects of miR-33a-5P on ABCA1/G1-mediated cholesterol efflux under inflammatory stress in THP-1 macrophages. PLoS One. (2014) 9(10):e109722. 10.1371/journal.pone.0109722 25329888 PMC4201478

[74] Wang LL Liu Y Chung JJ Wang T Gaffey AC Lu M Sustained miRNA delivery from an injectable hydrogel promotes cardiomyocyte proliferation and functional regeneration after ischemic injury. Nat Biomed Eng. (2017) 1:983–92. 10.1038/s41551-017-0157-y 29354322 PMC5773070

[75] Wai T García-Prieto J Baker MJ Merkwirth C Benit P Rustin P Imbalanced OPA1 processing and mitochondrial fragmentation cause heart failure in mice. Science. (2015) 350(6265):aad0116. 10.1126/science.aad0116 26785494

[76] Liu C Han Y Gu X Li M Du Y Feng N Paeonol promotes Opa1-mediated mitochondrial fusion via activating the CK2*α*-Stat3 pathway in diabetic cardiomyopathy. Redox Biol. (2021) 46:102098. 10.1016/j.redox.2021.102098 34418601 PMC8385203

[77] Xue Q Pei H Liu Q Zhao M Sun J Gao E MICU1 protects against myocardial ischemia/reperfusion injury and its control by the importer receptor Tom70. Cell Death Dis. (2017) 8(7):e2923. 10.1038/cddis.2017.280 28703803 PMC5550843

[78] Hu L Ding M Tang D Gao E Li C Wang K Targeting mitochondrial dynamics by regulating Mfn2 for therapeutic intervention in diabetic cardiomyopathy. Theranostics. (2019) 9(13):3687–706. 10.7150/thno.33684 31281507 PMC6587356

[79] Song Z Song H Liu D Yan B Wang D Zhang Y Overexpression of MFN2 alleviates sorafenib-induced cardiomyocyte necroptosis via the MAM-CaMKII*δ* pathway in vitro and in vivo. Theranostics. (2022) 12(3):1267–85. 10.7150/thno.65716 35154486 PMC8771548

[80] Ding M Shi R Cheng S Li M De D Liu C Mfn2-mediated mitochondrial fusion alleviates doxorubicin-induced cardiotoxicity with enhancing its anticancer activity through metabolic switch. Redox Biol. (2022) 52:102311. 10.1016/j.redox.2022.102311 35413642 PMC9006862

[81] Hu Y Li T Wang Y Li J Guo L Wu M Tollip attenuated the hypertrophic response of cardiomyocytes induced by IL-1beta. Front Biosci. (2009) 14(7):2747–56. 10.2741/3411 19273233

